# Immature instars of three species of *Rhodnius* Stål, 1859 (Hemiptera, Reduviidae, Triatominae): morphology, morphometry, and taxonomic implications

**DOI:** 10.1186/s13071-022-05200-2

**Published:** 2022-03-18

**Authors:** Gustavo Lázari Cacini, Jader de Oliveira, Tiago Belintani, Éder dos Santos Souza, Nicoly Olaia, Mara Cristina Pinto, João Aristeu da Rosa

**Affiliations:** 1 Faculdade de Ciências Farmacêuticas, Universidade Estadual Paulista (Unesp), Rodovia Araraquara-Jaú km 1, Araraquara, SP 14801-902 Brazil; 2 Departamento de Epidemiologia, Faculdade de Saúde Pública, Laboratório de Entomologia em Saúde Pública, Universidade de São Paulo, Av. Dr. Arnaldo 715, São Paulo, SP Brazil; 3 Instituto de Biologia, Universidade Estadual de Campinas (Unicamp), Campinas, SP Brazil; 4 Departamento de Entomologia, Fundação de Medicina Tropical Heitor Vieira Dourado (FMT-HVD), Manaus, Amazonas Brazil

**Keywords:** Chagas disease, Vector, Rhodniini, Triatomines, *Rhodnius*

## Abstract

**Background:**

Among the 18 genera of the Triatominae subfamily, three stand out for their diversity and epidemiological importance: *Triatoma*, *Panstrongylus*, and *Rhodnius*. *Rhodnius* includes 21 species that can transmit *Trypanosoma cruzi* (the etiological agent of Chagas disease, also known as American trypanosomiasis) and *Trypanosoma rangeli*. The *Rhodnius prolixus* complex comprises seven species, including *Rhodnius marabaensis*, *Rhodnius prolixus*, and *Rhodnius robustus*, which occur in the northern region of Brazil. Since both adults and immatures can carry *T. cruzi*, in this study the five nymphal instars of the three species mentioned were dorsally characterized.

**Methods:**

Using microscopy, morphometrics, and geometric morphometrics, the present work measures and describes the morphological characters of the five nymphal instars of *R. marabaensis*, *R. prolixus*, and *R. robustus*.

**Results:**

The study enabled the characterization of all five nymphal instars, as well as the distinction between the three species in each of their instars.

**Conclusions:**

The morphological, morphometrics of the head, thorax, and abdomen and geometric morphometrics studies of the head enabled the specific distinction of these three species in all five instars.

**Graphical Abstract:**

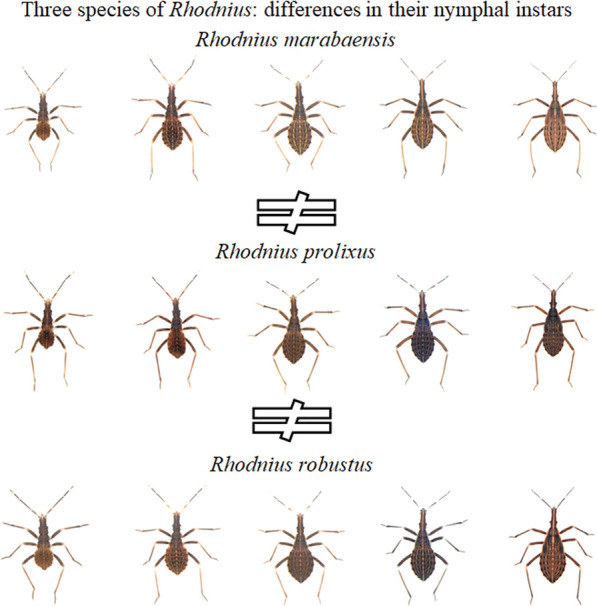

**Supplementary Information:**

The online version contains supplementary material available at 10.1186/s13071-022-05200-2.

## Background

Chagas disease is an endemic infection in the Americas caused by the protozoan *Trypanosoma cruzi* (Chagas, 1909) (Kinetoplastida, Trypanosomatidae) [1] and transmitted mainly by triatomines [2]. In South America, the Amazon region has a large potential to disseminate the disease, both for the relevant number of triatomine species living there and the difficulties related to vector surveillance and control [3]. In addition to the transmission by feces/urine infected with the protozoan, cases by oral transmission occurred due to the ingestion of food contaminated with *T. cruzi*, such as the juices of açaí (*Euterpe oleracea*), bacaba (*Oenocarpus bacaba*), jaci (*Attalea butyracea*), orange (*Citrus sinensis*), guava (*Psidium guajava*), sugarcane, and palm wine [3–5].

All species of Triatominae are potential or proven vectors of *T. cruzi* [6–8]. These species are placed into 18 genera, including *Rhodnius* Stål, 1859, which in addition to *T. cruzi* can transmit *Trypanosoma rangeli* Tejera, 1920 [9]. Although there is no evidence that *T. rangeli* is pathogenic to vertebrates, when examining or isolating Trypanosomatidae strains from triatomines, it is necessary to identify whether it is *T. cruzi* or *T. rangeli* [10]. *Rhodnius* has 21 species [11], of which 10 are found in northern Brazil: *Rhodnius amazonicus* Almeida, Santos & Sposina, 1973; *Rhodnius barretti* Abad-Franch et al., 2013; *Rhodnius brethesi* Matta, 1919; *Rhodnius milesi* Carcavallo et al., 2001; *Rhodnius montenegrensis* Rosa et al., 2012; *Rhodnius paraensis* Sherlock et al., 1977; *Rhodnius pictipes* Stål, 1872; *Rhodnius prolixus* Stål, 1859; *Rhodnius robustus* Larrousse, 1927, and *Rhodnius marabaensis* Souza et al., 2016 [12, 13].

Species belonging to *Rhodnius* present well-defined morphological characters that facilitate their identification in the Triatominae subfamily, but distinguishing them from one another is a complex task [2, 14]. The typical morphology of *Rhodnius* is characterized by the position of the antennal tubercle on the apex of the head and the absence of phallosome support in the genitalia of some species [2, 12, 14]. Their color tends towards dark/light brown, with spots that can be sharp [2]. Nymphs are characterized by an elongated head, antennal tubercles located in the distal one-third or one-fourth of the anteocular area, absence of ocelli, and spotted abdomen on the back. Median tubercles are located dorsally on the central longitudinal line from segment I–X [2, 9, 14–16]. Because of the related taxonomic difficulties and its epidemiological importance, *Rhodnius* is widely studied, yet its phylogeny has not been clarified and requires new studies [11, 17].

The tribe Rhodniini (*Rhodnius* + *Psammolestes* Bergroth, 1911) consists of a monophyletic group of two genera naturally occurring in the Neotropical region [18]. Arboreal habits are common in the genus, and most are associated with one or more palm species. Among the species studied in this work, *R. robustus* is found in Bolivia, Colombia, Ecuador, Peru, and Venezuela, as well as northern Brazil [2, 19]. In wild environments it is generally found in a variety of palm species, its presence having also been reported in domiciles and peridomiciles [20, 21]. This species is also related to food contamination and infection of forestry workers [22, 23]. *Rhodnius robustus* is very close to *R. montenegrensis*, but molecular studies have confirmed the specific status of each species [24, 25].

*Rhodnius marabaensis*, described in 2016 from the state of Pará, has a straw color, and its dorsal thorax has a trapezoidal shape limited by a straw carina. Its lobes usually show a black-spot pattern. The larger length of the second antennal segment and the keel-shaped head apex are two of the main morphological features of adults [12]. Recently, *R. marabaensis* had its specific status validated by transposable element analysis [25], as well as its biological cycle [26]. It is a species found in the wild with moderate epidemiological importance [12].

*Rhodnius prolixus* is considered the most important species in the transmission of Chagas disease in Venezuela, Colombia, and Central America [2, 19]. One of the factors that contribute to this is its optimal adaptation to human dwellings. It is similar to *R. robustus*, which makes the separation between them more difficult [27].

Taking all these considerations into account, this study aims to characterize *R. marabaensis*, *R. prolixus*, and *R. robustus* both morphologically and morphometrically, making it easier to distinguish the five nymphal instars of these three species. In addition to the epidemiological importance of the five nymphal instars, as they can carry *T. cruzi* and *T. rangeli* [9], the taxonomic validity of the study of their morphological characters must also be considered. Although the epidemiological importance of *R. marabaensis* is still unknown on account of its recent description, *R. prolixus* and *R. robustus* are important vectors of Chagas disease in the areas where they occur.

## Methods

### Specimens

**Fig. 1:**
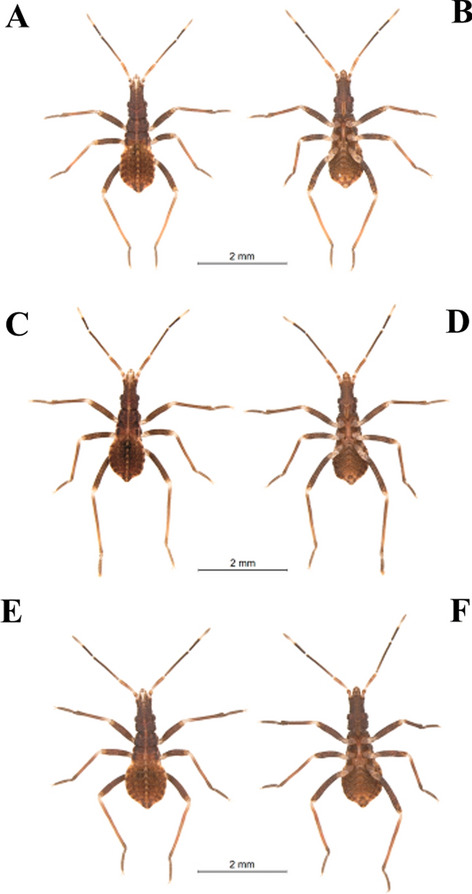
First-instar nymphs. *Rhodnius marabaensis*: **a** dorsal view, **b** ventral view; *R. prolixus*: **c** dorsal view, **d** ventral view; *R. robustus*: **e** dorsal view, **f** ventral view

**Fig. 2:**
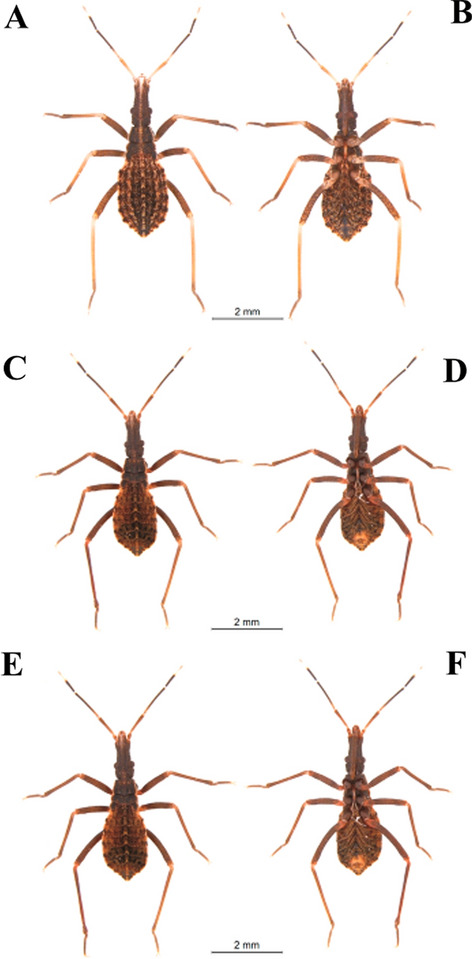
Second-instar nymphs. *R. marabaensis*: **a** dorsal view, **b** ventral view; *R. prolixus*: **c** dorsal view, **d** ventral view; *R. robustus*: **e** dorsal view, **f** ventral view

**Fig. 3:**
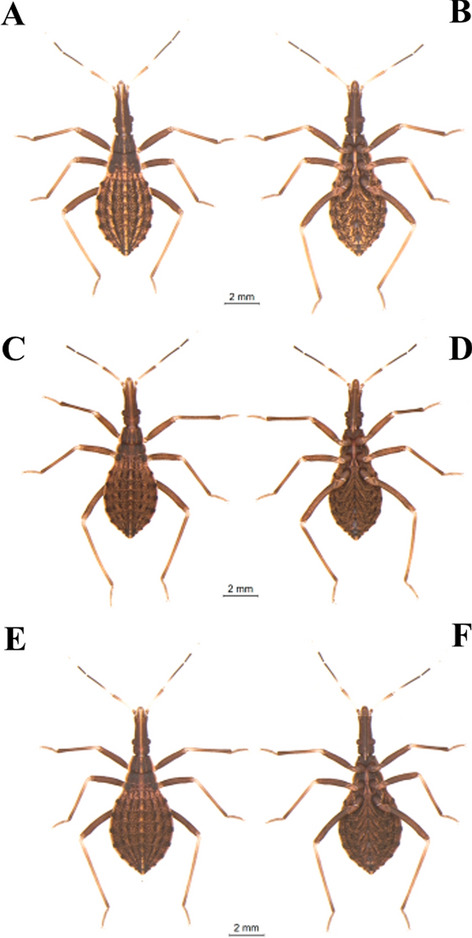
Third-instar nymphs. *R. marabaensis*: **a** dorsal view, **b** ventral view; *R. prolixus*: **c** dorsal view, **d** ventral view; *R. robustus*: **e** dorsal view, **f** ventral view

**Fig. 4:**
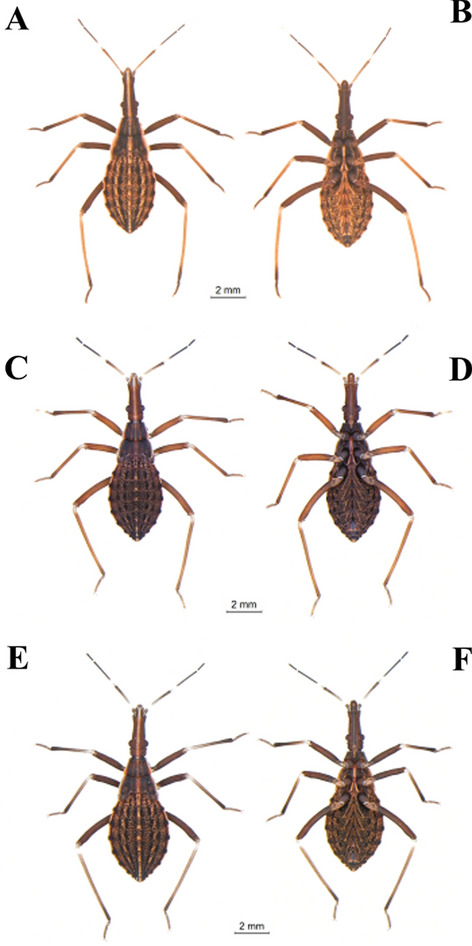
Fourth-instar nymphs. *R. marabaensis*: **a** dorsal view, **b** ventral view; *R. prolixus*: **c** dorsal view, **d** ventral view; *R. robustus*: **e** dorsal view, **f** ventral view

**Fig. 5:**
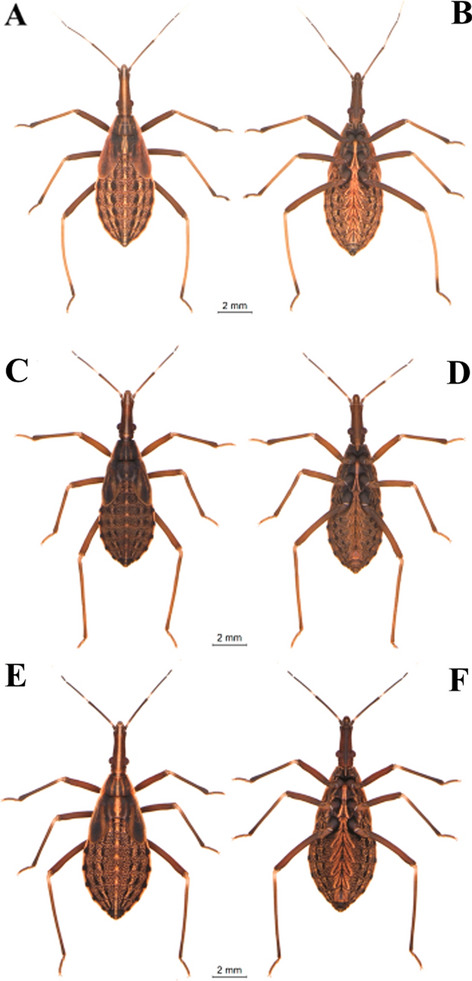
Fifth-instar nymphs. *R. marabaensis*: **a** dorsal view, **b** ventral view; *R. prolixus*: **c** dorsal view, **d** ventral view; *R. robustus*: **e** dorsal view, **f** ventral view

Specimens maintained in the Triatominae Insectarium (temperature 24 °C and 63% humidity) at the Faculty of Pharmaceutical Sciences of the São Paulo State University (Unesp-Araraquara) (https://www2.fcfar.unesp.br/#!/triatominae/) were used. *Rhodnius marabaensis* specimens (Figs. 1, 2, 3, 4, 5a, b) that originated the colony were collected in the county of Marabá, state of Pará, Brazil on May 12, 2014. The founders of the *R. prolixus* colony (Figs. 1, 2, 3, 4, 5c, d) were collected in Venezuela on May 23, 1983. The colony of *R. robustus* (Figs. 1, 2, 3, 4, 5e–f) originated from specimens collected on January 13, 2016, in the county of Ouro Preto do Oeste, state of Rondônia, Brazil. Nymphs of first, second, third, fourth, and fifth instars were taken from the respective colonies on the same day they were utilized. First-instar nymphs were selected right after egg hatching. Nymphs of second, third, fourth, and fifth instars were selected immediately after ecdysis (no abdominal distension from feeding). The nymphs were only fed (on ducks), every 7 days, to pass through the instars. The morphological and morphometric studies were conducted without verifying the gender distinction of the five nymphal instars.

### Morphological study

To generate images, five specimens of each of the five instars of *R. marabaensis*, *R. prolixus*, and *R. robustus* were used. Images of the dorsal sides of the head, thorax, and abdomen as well as the complete images of each of the five instars from dorsal view were obtained using a Leica M205 stereoscopic microscope and Leica Application Suite X software.

### Morphometric study

Fifteen specimens of first-, second-, third-, fourth-, and fifth-instar nymphs of *R. marabaensis*, *R. prolixus*, and *R. robustus* were measured using a Leica MZ APO stereoscopic microscope and the Motic Advanced 3.2 plus image analysis system.

The total length (TL), head length (HL), thorax length (XL), and abdomen length (AL) were measured for nymphs of all instars. Following Dujardin et al. [28], interocular (IO), anteocular (AO), and postocular (PO) distances were measured, as well as the three visible segments of the labium. The four antennal segments were also measured, according to Rosa et al. [29]. All measurements are expressed in millimeters.

The obtained data were analyzed by descriptive statistics, using *t*-tests for mean and standard deviation. Analysis of variance (ANOVA) and Tukey’s pairwise comparison were performed to evaluate the degree of differentiation of the three species using PAST software (Additional file 1).

### Geometric morphometrics of heads

Geometric morphometrics was used to evaluate variations in head shape and size using Cartesian reference coordinates. Variations among the heads of all nymphal instars of the studied species were evaluated. Fifteen heads of each instar were selected, and the images were obtained using a stereoscopic magnifying glass coupled to the Motic Advanced 3.2 plus scanning system. The coordinates of the reference points were selected according to Bookstein [30]. Four landmarks were adopted for the first and second instars and five landmarks for the other instars (Additional file 2). All of the landmarks are type 1 and were collected and processed using the modules available in the tpsDig v.1.18 software [31] and digitized using the CLIC package (https://xyom-clic.eu/the-clic-package/). Then the file with the raw coordinates was used for a generalized Procrustes analysis (GPA). GPA is a method that allows all the information related to size, position, and orientation of previously digitized anatomical frames to be eliminated [31]. The matrix of form was held in Euclidean space to generate a set of marks known as partial warps [30]. All the additional statistical forms were performed using Procrustes residues to analyze differences in the size and shape of the heads of each nymphal instar (Additional file 3). Procrustes ANOVA (*p* < 0.0001) [32] is used to infer differences between species. Procrustes ANOVA is a method for quantifying relative amounts of variation at different levels. These differences in size were assessed using an isometric estimator defined as centroid size (CS) [33]. Mahalanobis distances between pairs of species were calculated for measurements of shape and significance was assessed using a non-parametric test based on permutations (bootstrap, 10,000 replications) using MorphoJ [34]. In addition to that, using distance dice from Mahalanobis, neighbor-joining trees (NJ) were recovered using PAST v.3.25 [35]. To determine the relationships between species, canonical variable analysis (CVA) was performed using MorphoJ [34]. The CVA was performed associated with a resampling method (bootstrap, 10,000 replications) to build regions of trust in relation to the median size of the species center. A factorial map of the first two canonical fathers was created using MorphoJ, version 1.0.7a [34] (Additional file 3).

## Results

### Morphological description of the five nymphal instars of *R. marabaensis*, *R. prolixus*, and *R. robustus* by optical microscopy

**Fig. 6:**
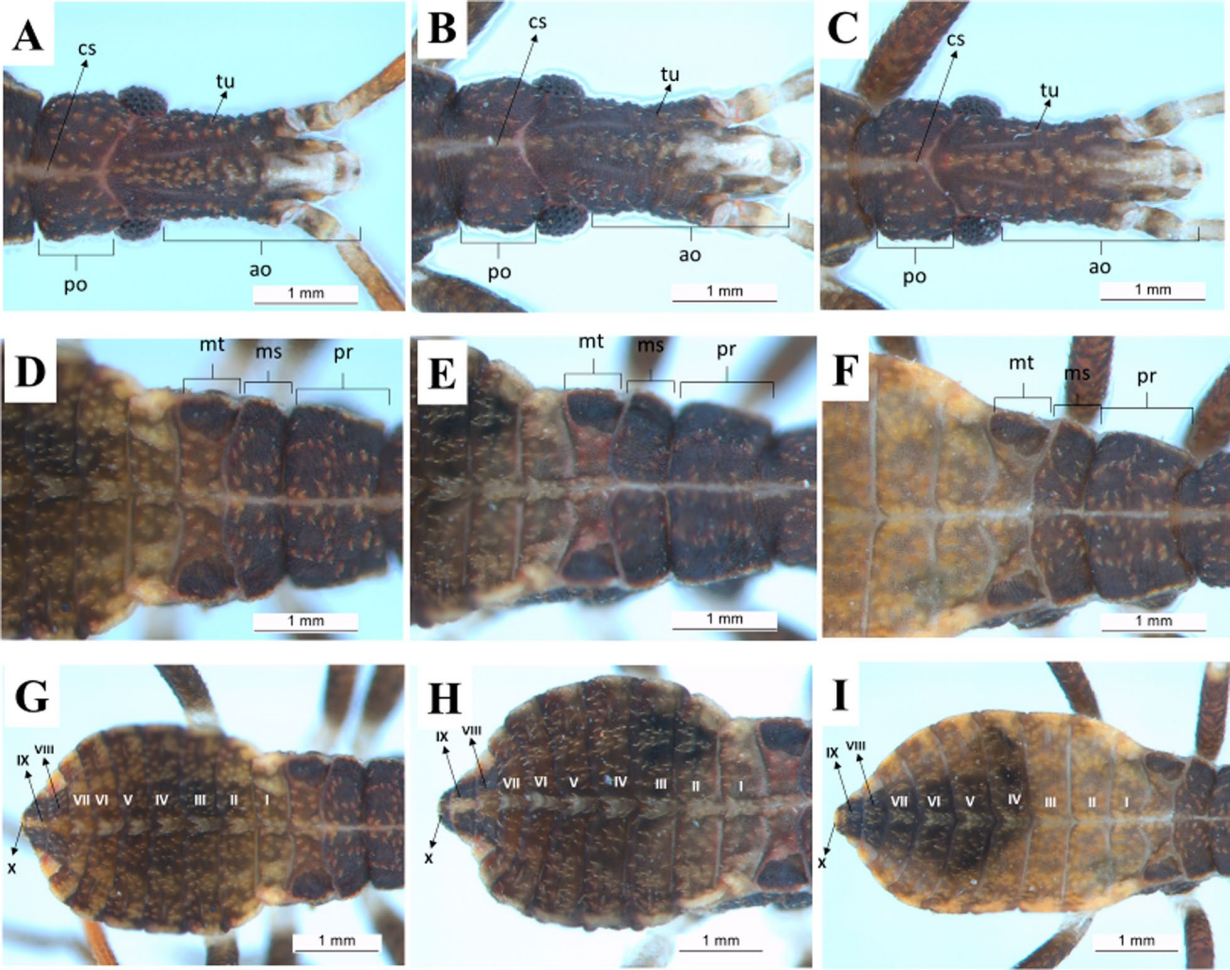
Dorsal view of first-instar nymphs. Head, thorax, and abdomen. **a**, **d**, **g**: *R. marabaensis*; **b**, **e**, **h**: *R. prolixus*; **c**, **f**, **i**: *R. robustus*; *cs* cephalic suture, *tu* tubercle, *ao* anteocular distance, *po* postocular distance, *mt* metanotum, *ms* mesonotum, *pr* pronotum, *I–X* abdominal segments

First instar: the head of the nymphs has a dark-brown cuticle covering all its granular extension due to the presence of tubercles with small setae, whose color is darker than that of the cuticle. The maxillary plate and mandibular plate showed no significant differences among the species. Regarding the postocular area, the species shows Y-shaped cephalic sutures (Fig. 6a–c). On the thorax, there are tubercles with setae in the three segments, located mainly in the center. The pronotum has a trapezoidal shape and is the segment with larger external borders, followed by the metanotum and the mesonotum. The three segments are well delimited by the dividing lines, but the line separating the mesonotum from the metanotum shows a sinuous protuberance that overlaps the metanotum in about one-third of its size (Fig. 6d–f). The abdomen of first-instar nymphs has a lighter color in comparison with the thorax and the head. There are many tubercles with setae lighter colored than the cuticle. Connexivum with darker spots along the margin surrounding the abdomen. A lighter median longitudinal stripe is evident all over the abdomen (Fig. 6g–i).

**Fig. 7:**
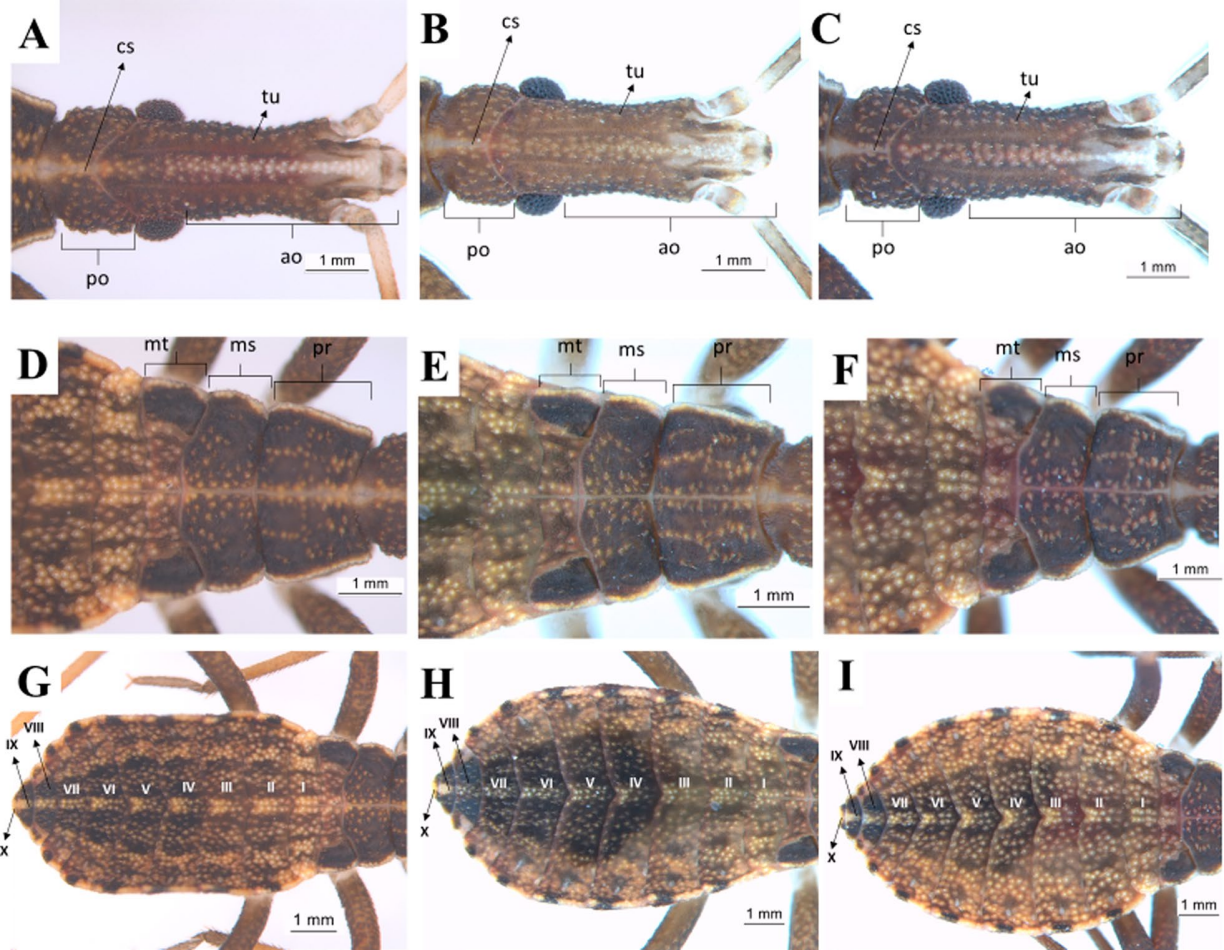
Dorsal view of second-instar nymphs. Head, thorax, and abdomen. **a**, **d**, **g**: *R. marabaensis*; **b**, **e**, **h**: *R. prolixus*; **c**, **f**, **i**: *R. robustus*; *cs* cephalic suture, *tu* tubercle, *ao* anteocular distance, *po* postocular distance, *mt* metanotum, *ms* mesonotum, *pr* pronotum, *I–X* abdominal segments

Second instar: the general aspects of the head of second-instar nymphs are similar to those described for first-instar nymphs. However, some differences are noticeable, such as the increase in the granulation grade and the number of setae in the three species, as well as the lighter color of the cuticle (Fig. 7a–c). Tubercles with setae are present in the three segments of the thorax, located mainly in the central portion. It is not possible to quantify the difference in size between the mesonotum and the metanotum, but, as in the first instar, the metanotum is broad on the sides and narrow in the central portion. The three segments are well delimited by the dividing lines, but the line separating the mesonotum from the metanotum has a sinuous protuberance that overlaps the metanotum in about one-third of its size (Fig. 7d–f). The abdomen has a median spot lighter than the cuticle in the dividing line of each of the urotergites, resembling a stripe. The connexival spots are more evident in this instar (Fig. 7g–i).

**Fig. 8:**
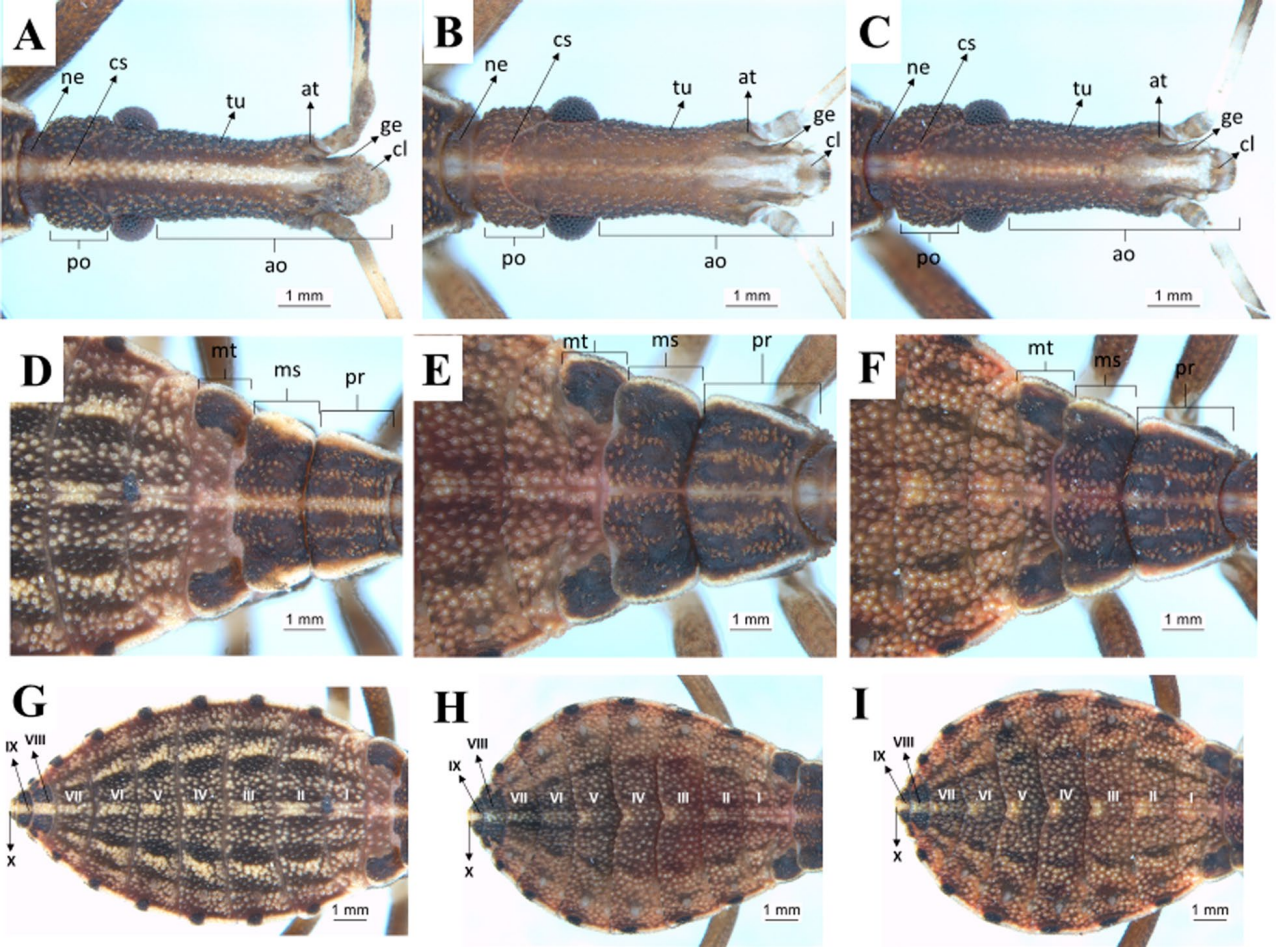
Dorsal view of third-instar nymphs. Head, thorax, and abdomen. **a**, **d**, **g**: *R. marabaensis*; **b**, **e**, **h**: *R. prolixus*; **c**, **f**, **i**: *R. robustus*; *ne* neck, *cs* cephalic suture, *tu* tubercle, *at* anteniferous tubercle, *ge* maxillary plate, *cl* clypeus, *ao* anteocular distance, *po* postocular distance, *mt* metanotum, *ms* mesonotum, *pr* pronotum, *I–X* abdominal segments

Third instar: for the three species, the maxillary plates are more rounded and extend until the end of the clypeus. Postocular cephalic sutures are also more rounded and roughly have a U-shape (Fig. 8a–c). Tubercles with setae are present in the three segments of the thorax, distributed across them. The pronotum has the shape of a trapezium and is the segment with the largest external borders, followed by the mesonotum and the metanotum. The three segments are well delimited by the dividing lines, but the line separating the mesonotum from the metanotum has a sinuous protuberance that overlaps the metanotum in about one-third of its size (Fig. 8d–f). The three species possess 2 + 2 dark stripes across the abdomen. An increase of spots in the connexivum can also be observed. In *R. marabaensis* abdominal segments III and IV are the broadest. The segments widen from segment I to III and gradually narrow from segment VI onwards. A central stripe of straw color is also easily visible on the abdomen of this species, and from the sides there is another straw-colored stripe, located between two black stripes. These three stripes are arched and have the same shape as the abdomen, but they are not continuous, as they are interrupted in the intersegmental sutures. In *R. prolixus* and *R. robustus* the largest abdominal segment is the IV (Fig. 8g–i).

**Fig. 9:**
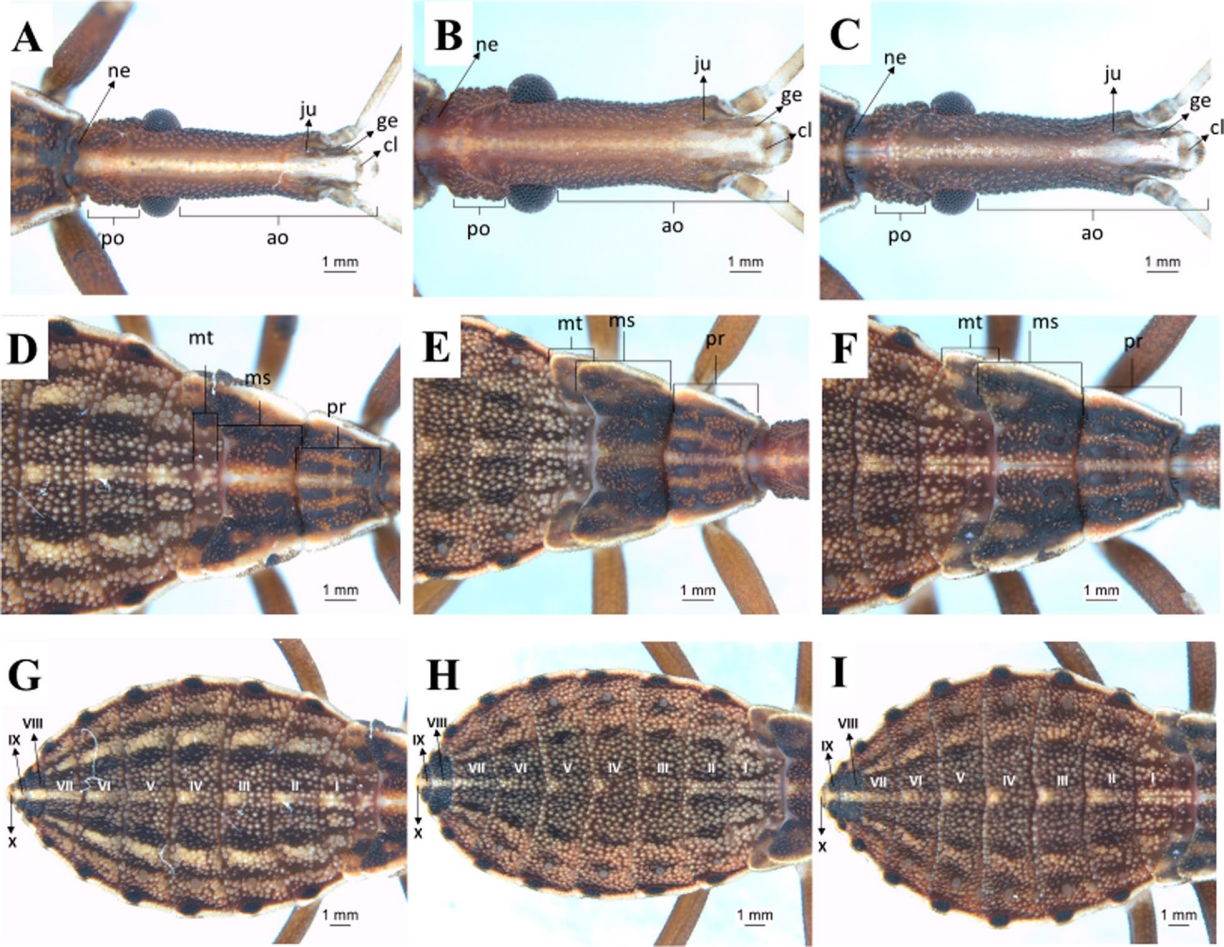
Dorsal view of fourth-instar nymphs. Head, thorax, and abdomen. **a**, **d**, **g**: *R. marabaensis*; **b**, **e**, **h**: *R. prolixus*; **c**, **f**, i: *R. robustus*; *ne* neck, *ju* mandibular plate, *ge* maxillary plate, *cl* clypeus, *ao* anteocular distance, *po* postocular distance, *mt* metanotum, *ms* mesonotum, *pr* pronotum, *I–X* abdominal segments

Fourth instar: fourth-instar nymphs present some peculiar characteristics, such as triangular mandibular plate, rounded maxillary plate surpassing the clypeus, and a higher granulation grade near the eyes (Fig. 9a–c). The three segments of the thorax have tubercles with setae. The mesonotum is the largest segment in this instar due to the presence of the first pair of wing pads. The second pair of wing pads originates from the metanotum. The three segments are well delimited by the dividing lines (Fig. 9d–f). In all three species the dark stripes on the abdomen are more evident, which gives the area a striped aspect. In this instar, the connexivum spots become more rounded. The central stripe on the abdomen has the same aspect as in the third instar and differentiates the three species: in *R. marabaensis* the three side stripes on the abdomen, a straw-colored stripe between two black ones to the right and left, are similar to what is observed in the third instar (Fig. 9g–i).

**Fig. 10:**
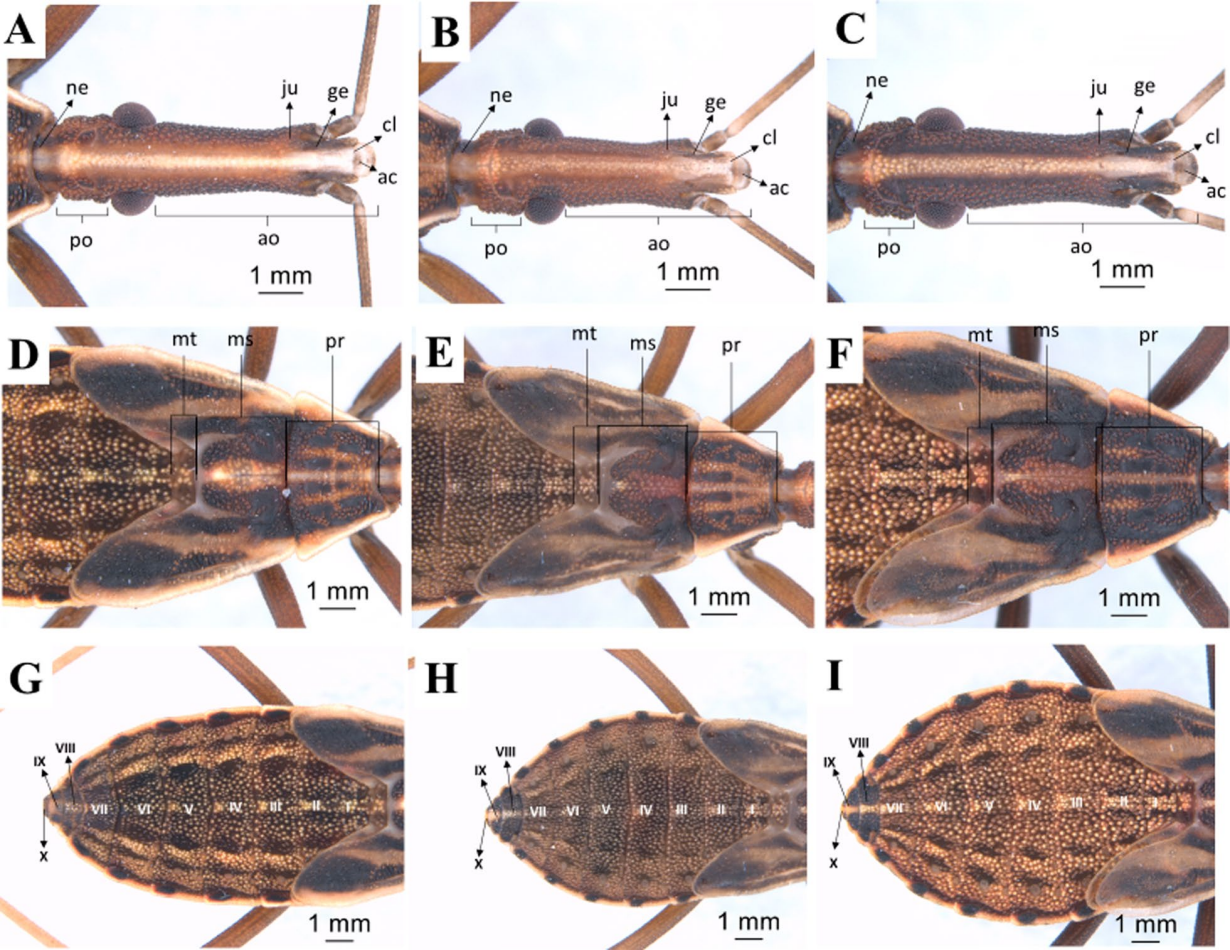
Dorsal view of fifth-instar nymphs. Head, thorax, and abdomen. **a**, **d**, **g**: *R. marabaensis*; **b**, **e**, **h**: *R. prolixus*; **c**, **f**, **i**: *R. robustus*; *ne* neck, *ju* mandibular plate, *ge* maxillary plate, *cl* clypeus, *ac* anteclypeus, *ao* anteocular distance, *po* postocular distance, *mt* metanotum, *ms* mesonotum, *pr* pronotum, *I–X* abdominal segments

Fifth instar: in this instar, all three species also have a quite visible white stripe on the head (Fig. 10a–c). There are tubercles with setae in the three segments of the thorax. The posterior pair of wing pads can be seen overlapping, projecting from the mesonotum through the anterior pair, which in turn projects from the mesonotum (Fig. 10d–f). It is possible to see only the central area of the metanotum as a result of this large overlapping. The anterior pair of wing pads reach the beginning of the third urotergite. In this instar, the central line of the abdomen retains the characteristics observed in the third and fourth instars. In *R. marabaensis* the three side stripes on the right and left (one straw and two black) observed in the third and fourth instars are still present. There is also an increase in the number of tubercles with setae (Fig. 10g–i).

In this study, morphological differences were also observed between the three species in their five nymphal instars (Table 1).

**Table 1:** Differences observed by optical microscopy between three species of *Rhodnius* in the five nymphal instars

Instars	Species	Characters		
		Head	Thorax	Abdomen
	*R. marabaensis*	–	–	Segments IV and V are the broadest. Segment I same length as the metanotum
First	*R. prolixus*	–	–	Larger, particularly between segments III and VI. Segment I same length as the metanotum
	*R. robustus*	–	–	Widens gradually from segment I to IV. Segment I longer than the metanotum
	*R. marabaensis*	–	Pronotum form of a trapezium. Mesonotum-metanotum dividing line straight	Straight external limits between segments III and VI
Second	*R. prolixus*	–	Longest pronotum. Mesonotum-metanotum dividing line concave	Maximum width in segment V
	*R. robustus*	–	Shortest pronotum. Mesonotum-metanotum dividing line straight	Widens gradually from segment I to IV
	*R. marabaensis*	Prominent white stripe from the antennal tubercle to the neck	Concavity is not seen	Stripe that has straw-colored marks elongated in each segment
Third	*R. prolixus*	The stripe is not so evident	Concavity near the metanotum	Such marks are neither so elongated nor clear
	*R. robustus*	Between the clypeus and the antennal tubercles; between the posterior region of the eye and the neck	Concavity is not seen	The marks are not elongated, yet clear
	*R. marabaensis*	White stripe is very clear	Pronotum-mesonotum line has a slight sinuosity overlapping a tiny part of the mesonotum	–
Fourth	*R. prolixus*	Stripe present, but narrower	Same as *R. marabaensis*	–
	*R. robustus*	Less clear in the intermediate portion	Pronotum-mesonotum line has a concave aspect, the same for the metanotum-abdomen line	–
	*R. marabaensis*	Maxillary plate reaches the clypeus (straight), the anteclypeus is curved	Side limit of the anterior wing pads is diffuse and broad	–
Fifth	*R. prolixus*	Maxillary plate surpasses the clypeus (curve), anteclypeus has a trapezoidal shape	Side limit of the anterior wing pads is a clear line	–
	*R. robustus*	Maxillary plate reaches the clypeus (concave), anteclypeus has a semicircular shape	Side limit of the anterior wing pads is a clear line	–

### Morphometric study of the five nymphal instars of *R. marabaensis*, *R. prolixus*, and *R. robustus*

With the acquired data it was possible to calculate the mean for each parameter and species, and then compare them to evaluate the degree to which the three *Rhodnius* species differ.

In the first and second instars, none of the parameters were statistically significant to evaluate the degree of the differences among the three *Rhodnius* species. As for the third instar, the parameter of the third segment of the antenna (*F*_(2,42)_ 23.12, *P* = 1.693) was significant (Table 2). In the fourth instar, only the postocular distance stands out (*F*_(2,42)_ 13.64, *P* = 2.718) (Table 3). Lastly, on the fifth instar, just the second segment of the antenna (*F*_(2,42)_ 36.32, *P* = 6.965) (Table 2) made it possible to evaluate the degree of the difference between *R. marabaensis*, *R. prolixus*, and *R. robustus*.

**Table 2:** Mean and standard deviation of the antennal and labium segments of three species of *Rhodnius*

Instars	Species	Characters						
		Antenna				Labium		
		1st seg	2nd seg	3rd seg	4th seg	1st seg	2nd seg	3rd seg
	*R. marabaensis*	0.14 ± 0.02^A^	0.36 ± 0.02^A^	0.69 ± 0.03^A^	0.64 ± 0.03^A^	0.17 ± 0.01^A^	0.52 ± 0.02^A^	0.29 ± 0.01^A^
First	*R. prolixus*	0.13 ± 0.01^A^	0.43 ± 0.02^B^	0.73 ± 0.03^B^	0.62 ± 0.03^A^	0.17 ± 0.01^AB^	0.54 ± 0.02^BC^	0.28 ± 0.01^AC^
	*R. robustus*	0.13 ± 0.01^A^	0.37 ± 0.02^A^	0.68 ± 0.03^A^	0.63 ± 0.05^A^	0.16 ± 0.01^AC^	0.53 ± 0.03^AC^	0.27 ± 0.03^BC^
	*R. marabaensis*	0.17 ± 0.01^A^	0.60 ± 0.03^A^	0.94 ± 0.03^A^	0.83 ± 0.06^A^	0.24 ± 0.02^A^	0.86 ± 0.03^A^	0.34 ± 0.01^A^
Second	*R. prolixus*	0.17 ± 0.01^AB^	0.68 ± 0.03^B^	0.95 ± 0.05^A^	0.78 ± 0.05^A^	0.26 ± 0.01^B^	0.86 ± 0.02^A^	0.37 ± 0.01^B^
	*R. robustus*	0.16 ± 0.01^AC^	0.59 ± 0.04^A^	0.93 ± 0.05^A^	0.79 ± 0.05^A^	0.24 ± 0.01^A^	0.81 ± 0.04^B^	0.35 ± 0.02^A^
	*R. marabaensis*	0.22 ± 0.01^A^	0.99 ± 0.05^A^	**1.29 ± 0.05**^**A**^	1.05 ± 0.08^A^	0.38 ± 0.03^A^	1.25 ± 0.08^A^	0.43 ± 0.02^A^
Third	*R. prolixus*	0.22 ± 0.01^A^	0.94 ± 0.03^B^	**1.16 ± 0.05**^**B**^	0.88 ± 0.08^B^	0.35 ± 0.03^B^	1.21 ± 0.07^AB^	0.45 ± 0.02^A^
	*R. robustus*	0.22 ± 0.01^A^	0.99 ± 0.03^A^	**1.24 ± 0.05**^**C**^	0.95 ± 0.10^B^	0.39 ± 0.01^A^	1.29 ± 0.10^AC^	0.44 ± 0.03^A^
	*R. marabaensis*	0.30 ± 0.01^A^	1.64 ± 0.15^A^	1.73 ± 0.14^A^	1.38 ± 0.16^A^	0.55 ± 0.04^A^	2.17 ± 0.15^A^	0.63 ± 0.05^A^
Fourth	*R. prolixus*	0.29 ± 0.02^A^	1.39 ± 0.09^B^	1.46 ± 0.13^B^	1.12 ± 0.08^B^	0.58 ± 0.07^AB^	1.95 ± 0.07^B^	0.64 ± 0.05^A^
	*R. robustus*	0.32 ± 0.01^B^	1.65 ± 0.10^A^	1.67 ± 0.10^A^	1.31 ± 0.09^A^	0.52 ± 0.06^AC^	1.87 ± 0.08^B^	0.54 ± 0.06^B^
	*R. marabaensis*	0.41 ± 0.02^A^	**2.71 ± 0.13**^**A**^	2.36 ± 0.09^A^	1.60 ± 0.23^A^	0.73 ± 0.05^A^	3.10 ± 0.15^A^	0.79 ± 0.04^A^
Fifth	*R. prolixus*	0.40 ± 0.01^A^	**2.17 ± 0.10**^**B**^	1.90 ± 0.15^B^	1.37 ± 0.12^BC^	0.74 ± 0.05^AC^	2.66 ± 0.06^B^	0.75 ± 0.07^AB^
	*R. robustus*	0.40 ± 0.02^A^	**2.48 ± 0.24**^**C**^	2.19 ± 0.29^A^	1.47 ± 0.21^AC^	0.79 ± 0.05^BC^	3.15 ± 0.15^A^	0.83 ± 0.03^AC^

Means with different superscripts at each site are significantly different from each other (one-way ANOVA followed by a Tukey test). In bold, we present statistically significant measurements for all three comparisons in Tukey’s pairwise test for differentiation of the species. Mean in millimeters

*Seg* segment

**Table 3:** Mean and standard deviation of the parameters of the three species of *Rhodnius*

Instars	Species	Characters						
		TL	HL	XL	AL	IO	AO	PO
	*R. marabaensis*	2.55 ± 0.08^A^	0.87 ± 0.02^A^	0.38 ± 0.01^A^	1.17 ± 0.07^A^	0.30 ± 0.01^A^	0.52 ± 0.02^A^	0.22 ± 0.02^A^
First	*R. prolixus*	2.66 ± 0.14^AB^	0.89 ± 0.03^AB^	0.37 ± 0.02^A^	1.29 ± 0.10^B^	0.29 ± 0.02^A^	0.55 ± 0.03^B^	0.21 ± 0.01^B^
	*R. robustus*	2.51 ± 0.15^AC^	0.85 ± 0.02^AC^	0.56 ± 0.02^A^	1.09 ± 0.11^A^	0.29 ± 0.03^A^	0.49 ± 0.03^A^	0.23 ± 0.02^A^
	*R. marabaensis*	4.49 ± 0.38^A^	1.24 ± 0.03^A^	0.65 ± 0.03^A^	2.22 ± 0.14^A^	0.36 ± 0.01^A^	0.81 ± 0.02^A^	0.26 ± 0.02^A^
Second	*R. prolixus*	4.56 ± 0.33^AB^	1.27 ± 0.04^AB^	0.68 ± 0.04^AB^	2.42 ± 0.19^B^	0.37 ± 0.02^A^	0.83 ± 0.03^AB^	0.26 ± 0.01^A^
	*R. robustus*	4.22 ± 0.36^AC^	1.20 ± 0.07^AC^	0.63 ± 0.05^AC^	2.11 ± 0.22^A^	0.34 ± 0.02^B^	0.78 ± 0.06^AC^	0.25 ± 0.02^A^
	*R. marabaensis*	6.53 ± 0.30^A^	1.77 ± 0.07^A^	0.96 ± 0.05^A^	3.21 ± 0.20^A^	0.43 ± 0.03^A^	1.13 ± 0.05^A^	0.37 ± 0.02^A^
Third	*R. prolixus*	6.63 ± 0.26^AC^	1.62 ± 0.05^B^	0.99 ± 0.04^AC^	3.45 ± 0.34^A^	0.45 ± 0.02^AC^	1.16 ± 0.03^A^	0.33 ± 0.02^B^
	*R. robustus*	6.84 ± 0.39^B^	1.81 ± 0.09^A^	1.02 ± 0.06^BC^	3.44 ± 0.19^A^	0.46 ± 0.02^BC^	1.16 ± 0.06^A^	0.33 ± 0.02^B^
	*R. marabaensis*	10.19 ± 0.46^A^	2.74 ± 0.16^A^	1.78 ± 0.12^A^	5.13 ± 0.34^A^	0.63 ± 0.03^A^	1.94 ± 0.12^A^	**0.51 ± 0.03**^**A**^
Fourth	*R. prolixus*	10.51 ± 0.40^A^	2.60 ± 0.09^BC^	1.78 ± 0.08^A^	5.31 ± 0.28^A^	0.65 ± 0.02^A^	1.84 ± 0.06^B^	**0.47 ± 0.02**^**B**^
	*R. robustus*	10.57 ± 0.54^A^	2.69 ± 0.12^AC^	1.83 ± 0.08^A^	5.31 ± 0.44^A^	0.65 ± 0.03^A^	1.93 ± 0.08^A^	**0.49 ± 0.02**^**C**^
	*R. marabaensis*	13.63 ± 0.50^A^	3.88 ± 0.19^A^	2.95 ± 0.15^A^	7.34 ± 0.40^A^	0.82 ± 0.04^A^	2.72 ± 0.12^A^	0.67 ± 0.05^A^
Fifth	*R. prolixus*	13.33 ± 0.40^A^	3.57 ± 0.16^B^	2.86 ± 0.13^AB^	7.31 ± 0.39^A^	0.82 ± 0.03^AC^	2.48 ± 0.11^B^	0.59 ± 0.02^B^
	*R. robustus*	13.39 ± 0.70^A^	3.94 ± 0.19^A^	3.01 ± 0.16^AC^	7.39 ± 0.49^A^	0.86 ± 0.03^BC^	2.73 ± 0.14^A^	0.65 ± 0.02^A^

Means with different superscripts at each site are significantly different from each other (one-way ANOVA followed by a Tukey test). In bold, we present statistically significant measurements for all three comparisons in Tukey’s pairwise test for differentiation of the species. Mean in millimeters

*TL* total length, *HL* head length, *XL* thorax length, *AL* abdomen length, *IO* interocular distance, *AO* anteocular distance, *PO* postocular distance

### Geometric morphometrics of the five nymphal instars of *R. marabaensis*, *R. prolixus*, and *R. robustus*

**Fig. 11:**
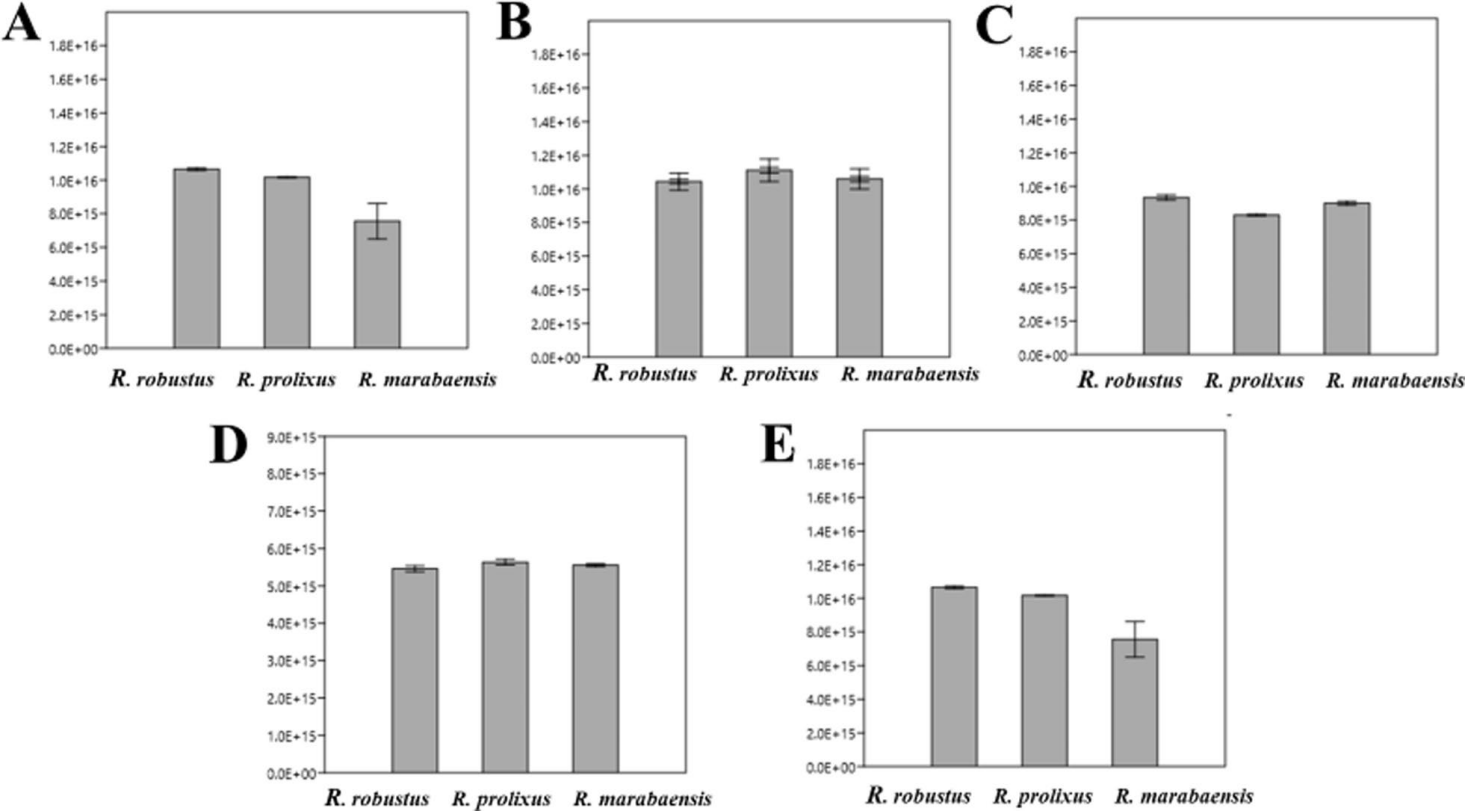
Geometric morphometry-based boxplot of centroid sizes (in pixels) among *R. marabaensis*, *R. prolixus*, and *R. robustus*. The thick black bar shows the standard error. **a** first instar; **b** second instar; **c** third instar; **d** fourth instar; **e** fifth instar

**Fig. 12:**
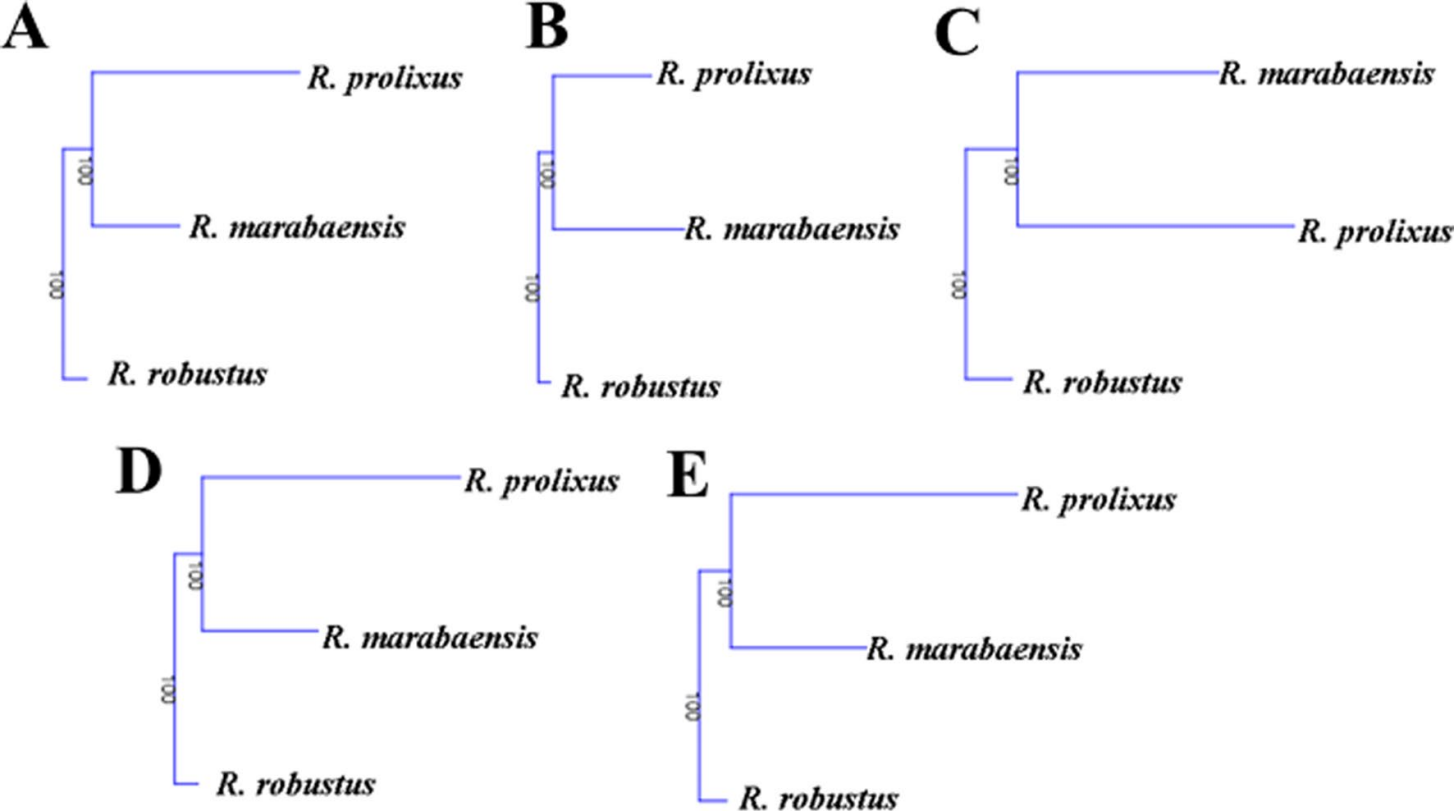
Neighbor-joining (NJ) tree generated from the measurements of Mahalanobis for the five nymphal instars of *R. marabaensis*, *R. prolixus*, and *R. robustus* (boot number = 100). **a** first instar; **b** second instar; **c** third instar; **d** fourth instar; **e** fifth instar

By ontogenetic geometric morphometry of the heads of nymphs, it was possible to describe the differences in shape and size of the five instars of *R. robustus*, *R. prolixus*, and *R. marabaensis*. CS measures show variability in the head size of the species. Furthermore, by the isometric measurement of the CS, the size gain among immature shapes can be clearly seen (Fig. 11). Analysis of the CS shows that differences among the size means are significant (*p* < 0.0001, supplementary material). *Rhodnius robustus* and *R. prolixus* have larger means than *R. marabaensis* (Fig. 11). Differences can also be explained as a percentage of the total variance among groups in the eigenvalues (auto values), the percentages being 89% for the first instar, 83% for the second, 98% for the third, 93% for the fourth, and 90% for the fifth. Mahalanobis distance was used as a metric estimator. The estimator considers the variations and correlations among groups defined a priori and enables pairwise comparison. Mahalanobis distances were significant among the pairs of the assessed species (*p* < 0.001, supplementary material). Dendrograms were built based on the values recovered for Mahalanobis distances and neighbor joining (NJ). The topology is identical for all instars (Fig. 12). It was possible to delimit the proximity between *R. prolixus* and *R. marabaensis* (Fig. 12). Procrustes ANOVA test also recovered significant values, showing shape differences among the species (*p* < 0.0001, supplementary material).

**Fig. 13:**
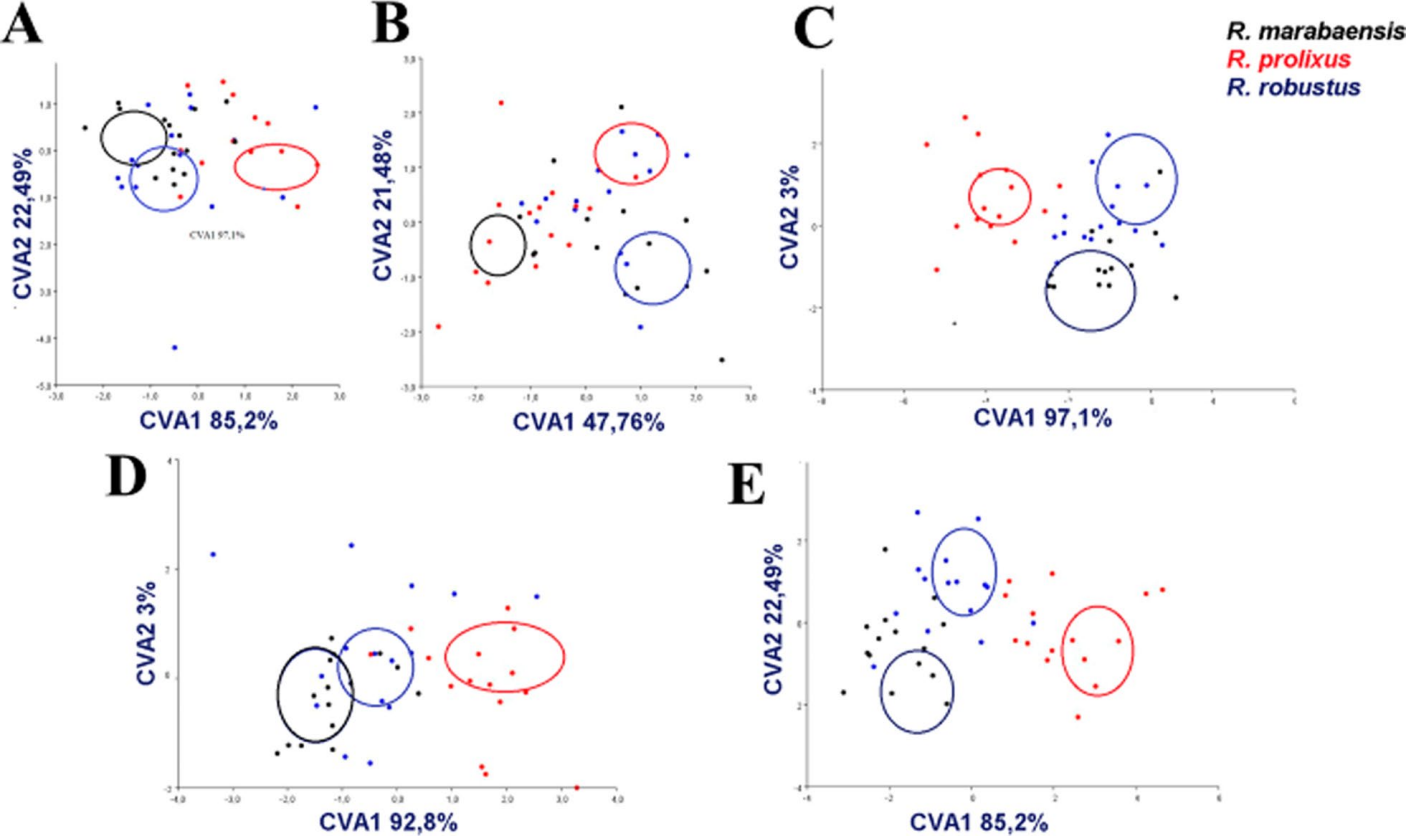
Scatter plots of the canonical variate analysis (CVA) of the grouped matrices for geometric morphometric of heads. The scores of the first canonical variable (CVA1) are on the *x*-axis and the scores for the second canonical variable (CVA2) are on the *y*-axis. The ellipses represent the confidence for means limits of each population (probability 0.5). (*R. marabaensis*—black ellipses; *R. prolixus*—red ellipses; *R. robustus*—blue ellipses). **a** first instar; **b** second instar; **c** third instar; **d** fourth instar; **e** fifth instar

The projection of the three species in the space defined by canonical axes 1 (CVA1) and 2 (CVA2) provides a description of the specified groups in the set of multivariate data. The analyses of the canonical variables resulted in 10 variables and explain 100% of the discrimination among the species (Fig. 13). The first two variables (CVA1 and CVA2) generated the following percentages: 85.2% and 22.49% for the first instar; 47.76% and 21.48% for the second; 97.1% and 3% for the third; 92.8% and 3% for the fourth; and 85.2% and 22.49% for the fifth (Fig. 13). The grouping in the space of the canonical axis shows an overlapping relationship between *R. prolixus*, *R. robustus*, and *R. marabaensis* in the first and fourth instar; however, the separation of populations in the second, third, and fifth instars is clear. *R. marabaensis* is the species that was best separated in the CVA analysis.

## Discussion

A striking feature of Triatominae is that males, females, and nymphs of all instars can transmit *T. cruzi* if infected [36, 37]. In several field collections carried out between 1989 and 2021 by Rosa et al. [unpublished data], a higher prevalence of nymphs than adults was observed. Thus, studies regarding the specific identification of nymphal instars become necessary, which is the objective of this study. Therefore, studies about nymphal instars not only have taxonomic and phylogenetic interest, but also have epidemiological importance. Specifically, about the genus *Rhodnius*, the following works can be mentioned: Mascarenhas [38], which studied the five instars of *R. brethesi*; Ponsoni et al. [39] and Marconato et al. [40], which carried out a biometric study of nymphs of *R. neglectus* Lent, 1954 and *R. prolixus*; and Santos [41], which described nymphs of the five instars of *Rhodnius colombiensis* Meija, Galvão & Jurberg, 1999, *Rhodnius ecuadoriensis* Lent & León, 1958, *R. milesi*, and *Rhodnius stali* Lent, Jurberg & Galvão, 1993.

Morphological characters are useful tools for taxonomic and systematic studies in Triatominae, in addition to being useful for epidemiological surveillance. The morphological analyses showed the separation of the three species by characters observed on the head, thorax, and abdomen shape. This made it possible to separate them in all five nymphal instars and to characterize for the first time the development instars of *R. marabaensis*. In their chapter about nymphal instars, Lent and Wygodzinsky [2] mentioned that *R. prolixus* and *R. robustus* do not have sub median tubercles or aggregations of granules along the midline, but such characters were observed in all five nymphal instars of those species, as well as in *R. marabaensis*. Rosa et al. [37], studying first- and second-instar nymphs of *Triatoma wygodzinskyi* Lent, 1951, distinguished the two instars by morphological characters of the thorax. Thus, by scanning electronic microscopy, they observed the absence of collar, glabrous areas, and tubercles in the first instar of *T. wygodzinskyi*, which were present in the second instar. Nevertheless, the differentiation among third, fourth, and fifth instars of *R. marabaensis*, *R. prolixus*, and *R. robustus* was made using the same characters observed by Rosa et al. [37] in nymphs of the previously mentioned instars of *T. wygodzinskyi*, i.e., the formation and conformation of the two pairs of wing pads located on the thorax. Although the ventral surface was not described, it is very likely that in future studies, new differential characters between the three species will be added to those already described in this work.

In this study, the results of the morphometry of characters from the abdomen, antenna, head, labium, and thorax showed little discrimination between the three species. In general, the compared averages are small or not significant, and the morphometric study is not suitable for identification. However, *R. marabaensis* had its nymphs characterized morphometrically and morphologically for the first time.

The relative length of the four antennal segments in *R. marabaensis* showed the same pattern for the first three instars, another for the fourth instar, and a third pattern for the fifth instar, whereas *R. prolixus* and *R. robustus* showed the same pattern for the first and second instars, another for the third and fourth instars, and a third one for the fifth instar. Santos [41], measuring *R. colombiensis*, *R. ecuadoriensis*, and *R. stali*, found two patterns of relative length for antennal segments of the five nymphal instars. For *R. milesi*, the author found three patterns, one for the first and second instars, another for the fourth and fifth instars, and a third one for the third instar, hence different patterns from the ones observed in *R. marabaensis*, *R. prolixus*, and *R. robustus*.

Rosa et al. [42] carried out a morphometric study of the four antennal segments of nymphs of the five instars and adults of *Panstrongylus megistus* (Burmeister, 1835), *R. neglectus*, *R. prolixus*, and *Triatoma vitticeps* (Stål, 1859). The patterns identified in *R. neglectus* and *R. prolixus* were the same as those found for *R. prolixus* and *R. robustus* in this work. Rosa et al. [29] measured the antennal segments of *Triatoma rubrovaria* (Blanchard, 1843) and found patterns different from *R. marabaensis*, *R. prolixus*, and *R. robustus*, but similar to those observed in *P. megistus* by Rosa et al. [42]. However, in relation to the relative length of the four antennal segments, it is not possible to differentiate the studied species. The different results were described for *R. colombiensis*, *R. ecuadoriensis*, *R. milesi*, *R. stali* [41], and *R. neglectus* [42], *T. rubrovaria* [29], *P. megistus*, and *T. vitticeps* [42]. Furthermore, our data show that *R. prolixus* and *R. robustus* are like *R. neglectus* [42] and can be distinguished from *R. colombiensis*, *R. ecuadoriensis*, *R. milesi*, and *R. stali* [41] as well as *T. rubrovaria* [29], *P. megistus*, and *T. vitticeps* [42] for this characteristic.

Geometric morphometry enables the evaluation of the variation in shape in relation to causal effects [43]. The technique allows us to quantify biological forms and discuss the evolution of phenetic patterns [34]. The technique is used in paleontological, anthropological, ecological, zoological, and botanical studies [30, 34]. In triatomines, geometric morphometry is used to assess the shape and size variables of hemelytra [44, 45], heads [13, 46], and eggs [47]. It is also used for ontogenetic studies [48–50].

Recently two subcomplexes of the genus *Triatoma* Laporte, 1832 were studied using geometric morphometrics, which indicated the potential of the technique to study specimens that are phylogenetically close [44, 46]. Geometric morphometrics allowed the differences in head shape and size of the five nymphal instars to be described. In relation to the CS, all values obtained were significant and enabled the differentiation of the three species in the five nymphal instars. Variation was observed among the instars, but considering the general aspect, *R. robustus* was easily characterized by the geometric profile of the heads of nymphs. The second and fourth instar showed less discrimination potential, i.e., only approximated size means were recovered.

The metric estimator of Mahalanobis distance was used to recover NJ dendrograms, where it is possible to visualize that in all evaluated instars *R. robustus* is distant, whilst *R. prolixus* and *R. marabaensis* form a single clade. However, CVA ellipses showed that in the first and second instars, *R. marabaensis* and *R. robustus* remain close, while groups are clearly separated in the third, fourth, and fifth instars. Regarding the shape, the values of the Procrustes ANOVA test revealed differences among the cephalic capsules, enabling discrimination. It was shown that the multivariate morphometric technique is more efficient for discriminating against the studied species when confronted with linear morphometric data.

## Conclusion

In this study, the morphological and morphometric differences in three *Rhodnius* species were evaluated. New data were also provided for *R. marabaensis*. Furthermore, it was shown that the morphology of the head (third, fourth, and fifth), thorax (second and fifth instar), and abdomen (first, second, third, and fifth instar) are useful in discriminating the studied species. Through morphometric analysis of the head, it was verified that the postocular distance of the fourth instar and the lengths of the antennal segments of the third and fifth instars distinguish the three species. Lastly, geometric morphometry proved to be useful for these species. The size and shape variables clearly show the differences between *R. marabaensis*, *R. prolixus*, and *R. robustus*.

## Supplementary Information

**Additional file 1.** Morphometry tests.

**Additional file 2.** Landmarks of heads.

**Additional file 3.** Geometric morphometrics.

## Data Availability

“Belintani, Tiago (2021), “Immature instars of three species of Rhodnius (Hemiptera: Reduviidae: Triatominae): morphological and morphometric studies”, Mendeley Data, V5, https://doi.org/10.17632/tjgj87yw78.5.
