# Supplementary material for: Immature instars of three species of Rhodnius Stål, 1859 (Hemiptera, Reduviidae, Triatominae): morphology, morphometry, and taxonomic implications
Source: Parasit Vectors. 2022 Mar 18;15:91. doi: 10.1186/s13071-022-05200-2 (PMC8932165; doi:10.1186/s13071-022-05200-2)

1. R. marabaensis, B- R. prolixus, C- R. robustus

Abdomen


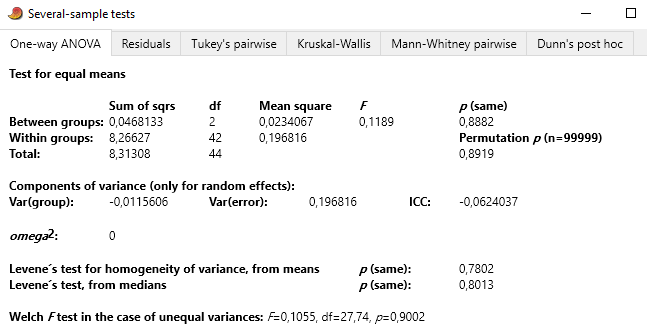


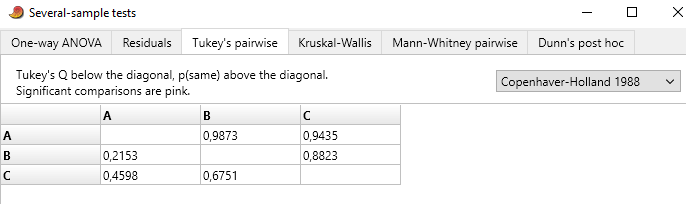


Total length


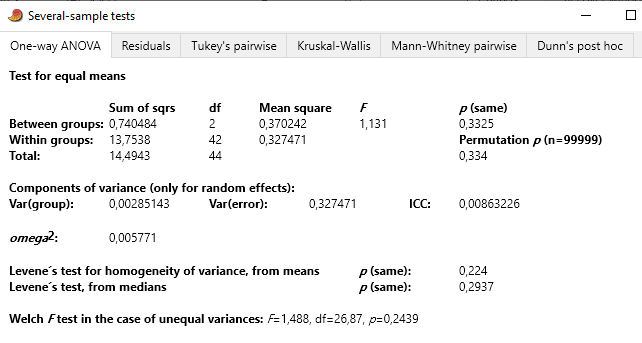


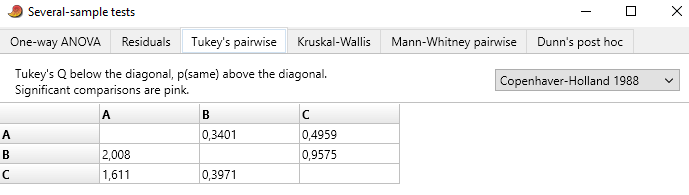


Thorax


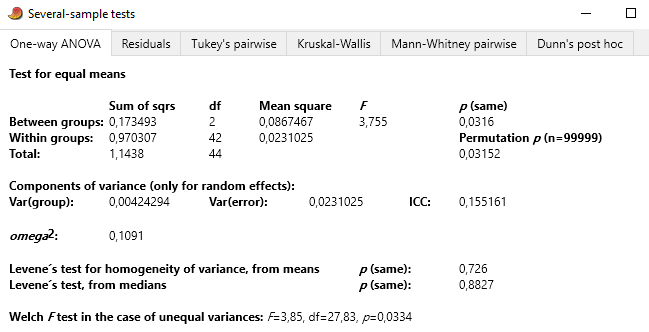


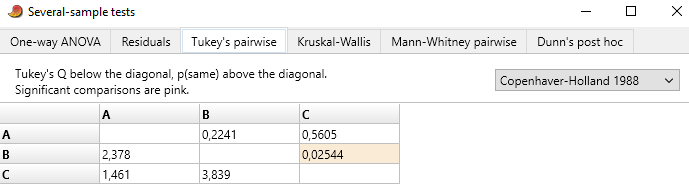


Head length


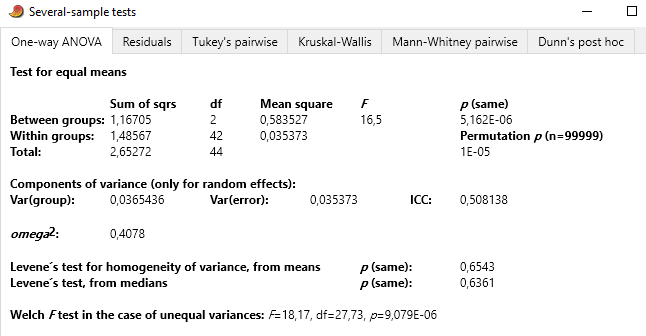


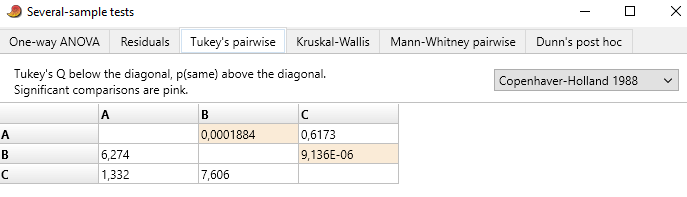


Interocular distance


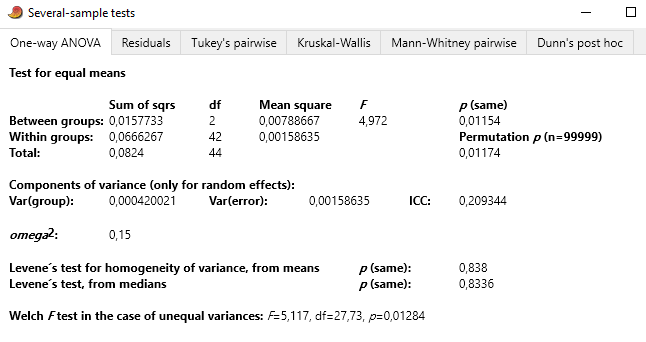


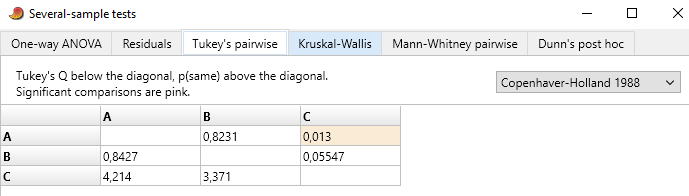


Anteocular distance


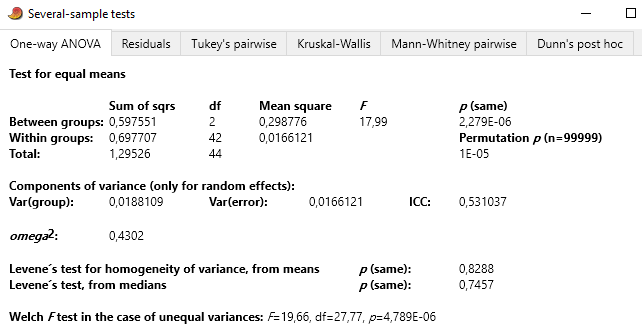


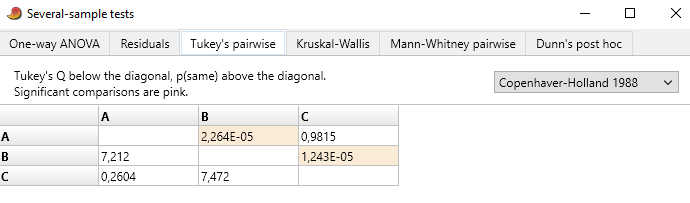


Postocular distance


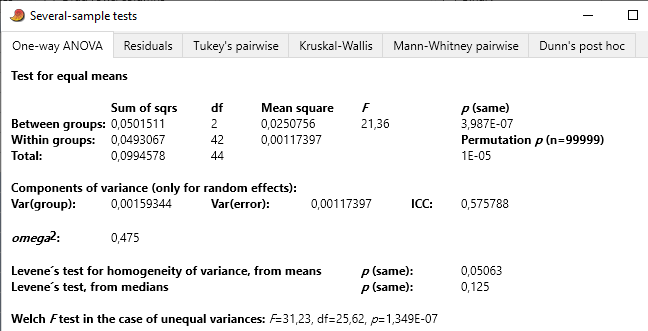


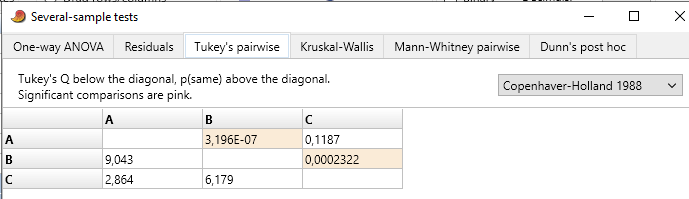


1st segment proboscis


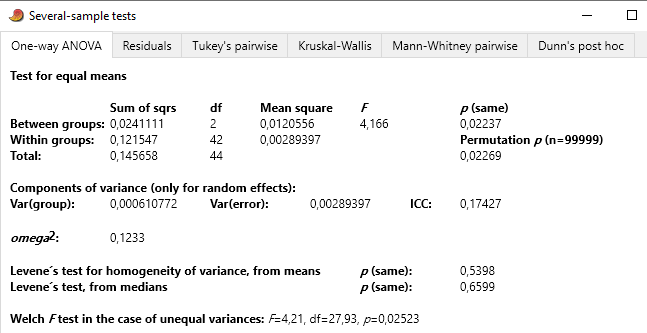


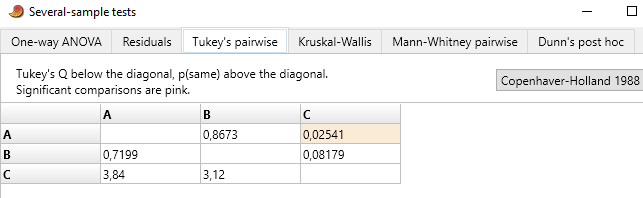


2nd segment proboscis


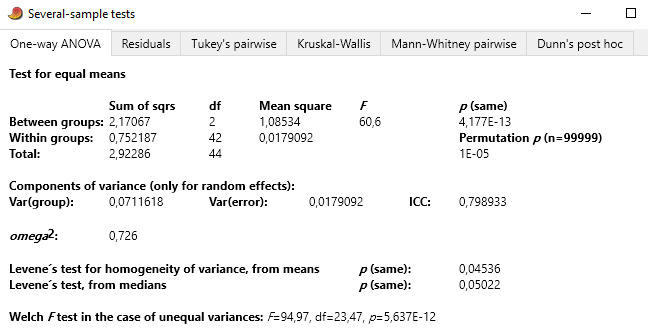


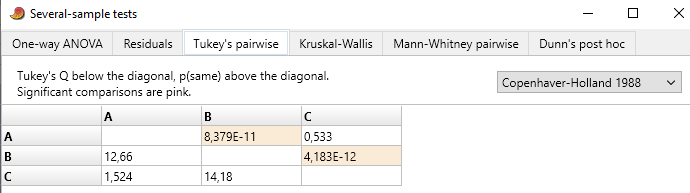


3rd segment proboscis


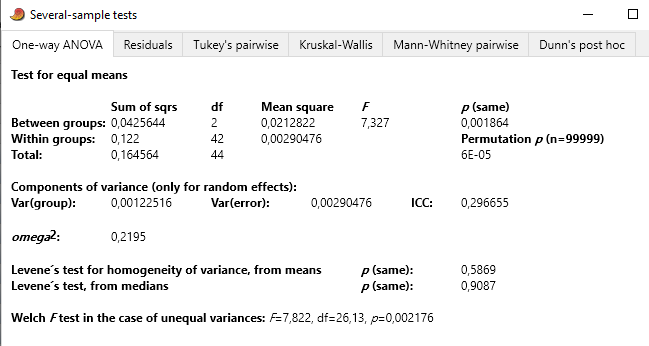


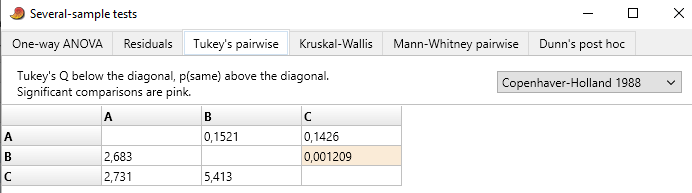


1st segment antenna


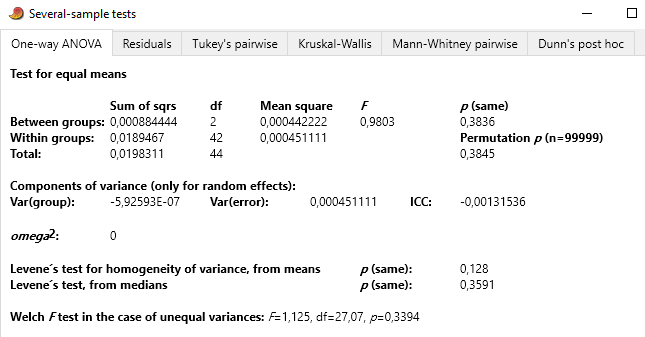


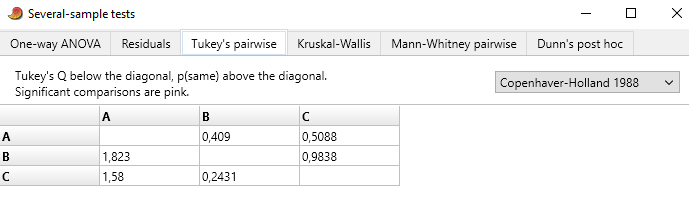


2nd segment antenna


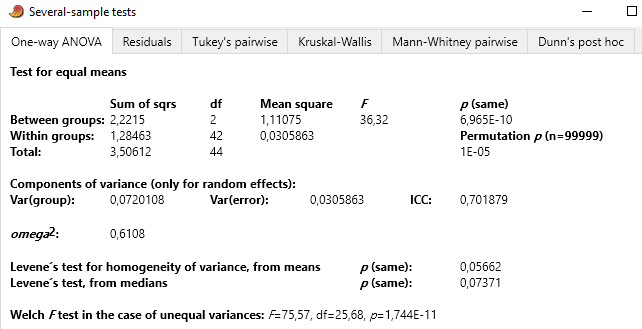


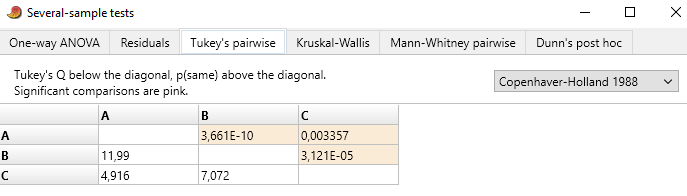


3rd segment antenna


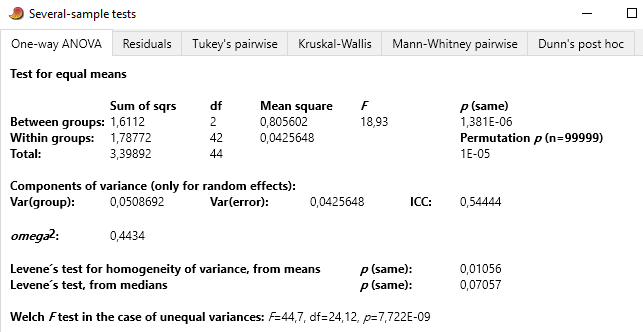


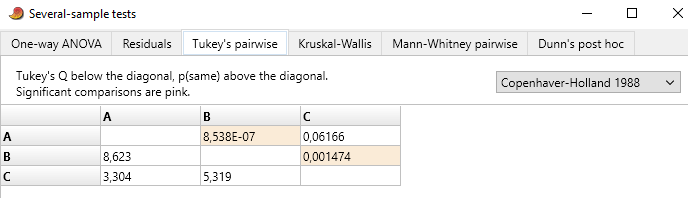


4th segment antenna


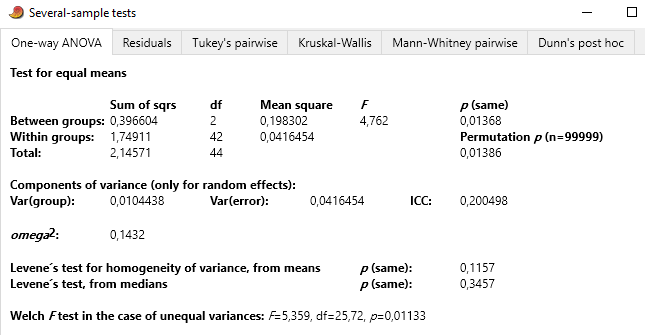


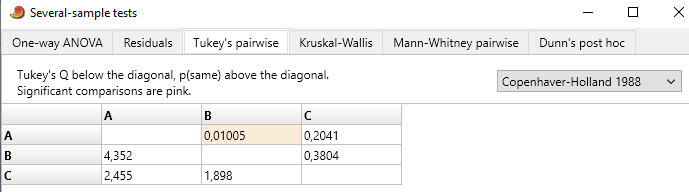

Supplement: Supplementary file 1 — Additional file 1. Morphometry tests. [file 13071_2022_5200_MOESM1_ESM.zip › Additional file 1-Morphometry tests/5th instar.docx]
