# Supplementary material for: Immature instars of three species of Rhodnius Stål, 1859 (Hemiptera, Reduviidae, Triatominae): morphology, morphometry, and taxonomic implications
Source: Parasit Vectors. 2022 Mar 18;15:91. doi: 10.1186/s13071-022-05200-2 (PMC8932165; doi:10.1186/s13071-022-05200-2)
Supplement: Supplementary file 2 — Additional file 2. Landmarks of heads. [file 13071_2022_5200_MOESM2_ESM.pdf]

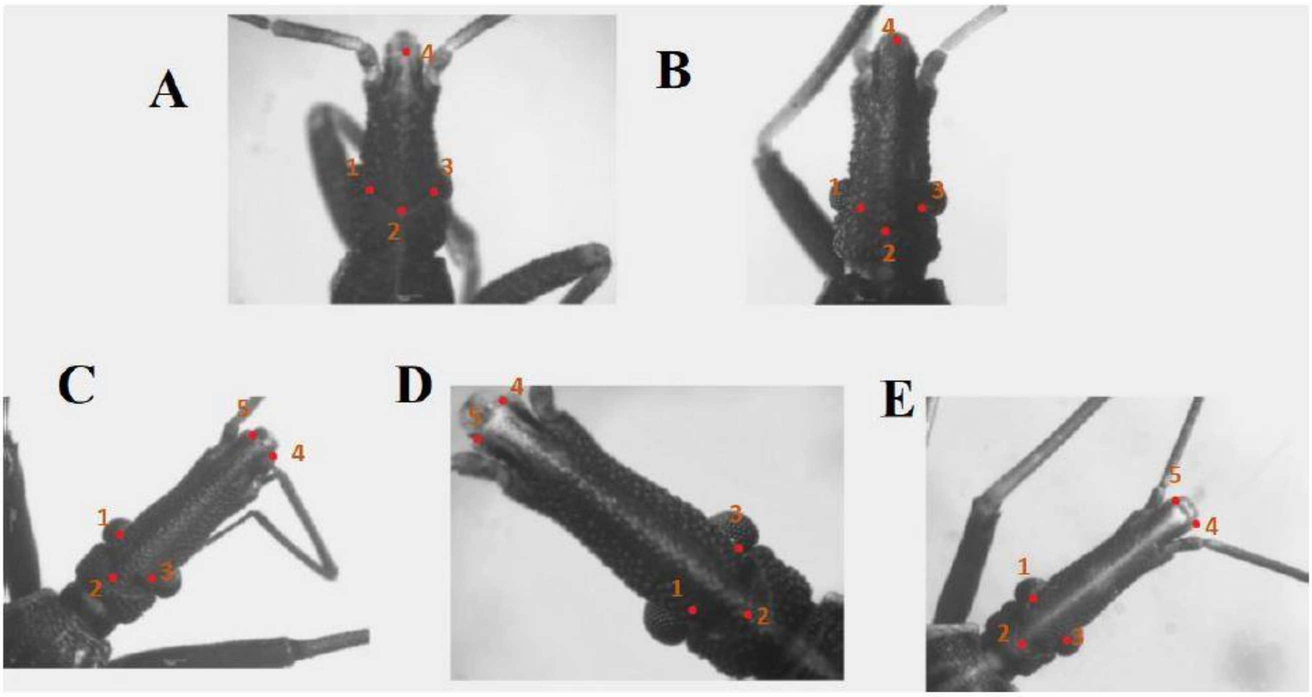

Additional file 2 - Landmarks adopted in the geometric morphometrics study of heads. **a** first-instar nymphs, **b** second-instar nymphs, **c** third-instar nymphs, **d** fourth-instar nymphs and **e** fifth-instar nymphs. The first and second instars nymphs were evaluated by four landmarks, the others instars were evaluated by five landmarks.
